# Predictive Value of Circulatory Total VEGF-A and VEGF-A Isoforms for the Efficacy of Anti-PD-1/PD-L1 Antibodies in Patients with Non-Small-Cell Lung Cancer

**DOI:** 10.3390/cancers17040572

**Published:** 2025-02-07

**Authors:** Tetsu Hirakawa, Kakuhiro Yamaguchi, Kunihiko Funaishi, Kiyofumi Shimoji, Shinjiro Sakamoto, Yasushi Horimasu, Takeshi Masuda, Taku Nakashima, Hiroshi Iwamoto, Hironobu Hamada, Shingo Yamada, Noboru Hattori

**Affiliations:** 1 Department of Molecular and Internal Medicine, Graduate School of Biomedical and Health Sciences, Hiroshima University, Hiroshima 734-8551, Japan; bgdwj032@yahoo.co.jp (T.H.); ta-masuda@hiroshima-u.ac.jp (T.M.); tnaka@hiroshima-u.ac.jp (T.N.); nhattori@hiroshima-u.ac.jp (N.H.); 2 Department of Physical Analysis and Therapeutic Sciences, Graduate School of Biomedical and Health Sciences, Hiroshima University, Hiroshima 734-8551, Japan; 3 Shino-Test Corporation, Sagamihara 252-0331, Japan

**Keywords:** vascular endothelial growth factor-A, VEGF_121_, non-small cell lung cancer, anti-PD-1/PD-L1 antibody, biomarker

## Abstract

Background/Objectives: Vascular endothelial growth factor (VEGF)-A promotes an immunosuppressive tumor microenvironment, potentially affecting the efficacy of anti-programmed cell death 1 (PD-1)/programmed cell death ligand 1 (PD-L1) antibody therapy. VEGF_121_ and VEGF_165_, VEGF-A isoforms, promote and inhibit tumor growth, respectively. Additionally, VEGF-A levels differ depending on whether they are measured in serum or plasma. However, whether the serum or plasma levels of total VEGF-A (tVEGF-A) or its isoforms are the most suitable for predicting anti-PD-1/PD-L1 antibody therapy efficacy remains unclear. Methods: Eighty-six patients with non-small-cell lung cancer (NSCLC) who were treated with anti-PD-1/PD-L1 antibody monotherapy between December 2015 and December 2023 were retrospectively enrolled. The association between the serum and plasma levels of tVEGF-A and its isoforms (VEGF_121_ and VEGF_165_) and treatment outcomes was analyzed. Results: The median progression-free survival (PFS) was 2.9 months, and the objective response rate (ORR) was 23.3%. PFS was significantly shorter in patients with higher tVEGF-A serum levels (≥484.2 pg/mL) than in those without (median PFS 2.1 vs. 3.7 months, *p* = 0.004). In contrast, plasma tVEGF-A levels could not be used to stratify PFS. Therefore, the serum levels of VEGF-A isoforms were measured. Patients with higher VEGF_121_ serum levels (≥523.5 pg/mL) showed both significantly shorter PFS (median PFS 2.3 vs. 3.3 months, *p* = 0.022) and a lower ORR (9.7% vs. 30.9%, *p* = 0.033) than those without. Multivariate Cox and logistic regression analyses showed that higher levels of serum VEGF_121_ were significantly associated with shorter PFS and a lower ORR. Conclusions: Serum VEGF_121_ levels may be useful in predicting anti-PD-1/PD-L1 antibody monotherapy efficacy.

## Simple Summary

Vascular endothelial growth factor (VEGF)-A is known to play a crucial role in the tumor microenvironment. This study investigated the relationship between circulating total VEGF-A (tVEGF-A) and its isoforms with the therapeutic effects of anti-programmed cell death 1 (PD-1)/programmed cell death ligand 1 (PD-L1) antibody monotherapy in patients with non-small-cell lung cancer (NSCLC). Higher levels of tVEGF-A were associated with shorter progression-free survival (PFS) in anti-PD-1/PD-L1 antibody monotherapy only when measured in serum, not in plasma. Notably, higher levels of serum VEGF_121_, an isoform of VEGF-A, were significantly associated with not only shorter PFS but also a lower objective response rate. Serum VEGF_121_ levels could serve as a useful biomarker for predicting anti-PD-1/PD-L1 antibody monotherapy efficacy in patients with NSCLC.

## 1. Introduction

Non-small-cell lung cancer (NSCLC) has a poor prognosis compared to many other cancers [1]. The prognosis of advanced NSCLC has dramatically improved with the advent of nivolumab [2,3], pembrolizumab [4,5] (anti-programmed cell death 1 [PD-1] antibodies), and atezolizumab [6] (anti-programmed cell death ligand 1 [PD-L1] antibodies). Currently, the PD-L1 tumor proportion score (TPS) is a predictor of the response to anti-PD-1/PD-L1 antibody therapy and is used in clinical practice [7]. However, even in patients with advanced NSCLC harboring high PD-L1 expression, the response rate for anti-PD-1/PD-L1 antibody monotherapy as a first-line treatment is only 38.3–44.8% [4,6], and the accuracy of predictions based on PD-L1 expression is limited. Therefore, there is an urgent need to identify new biomarkers other than PD-L1 that can predict the efficacy of anti-PD-1/PD-L1 antibody therapy.

Vascular endothelial growth factor (VEGF)-A is a homodimer protein of 40–45 kDa that is secreted by various cells, including tumor cells, immune cells, and platelets [8,9,10,11]. VEGF-A binds to vascular endothelial growth factor receptor (VEGFR) and neuropilin (NRP) [12]. VEGF-A expression is regulated by hypoxia-inducible factor-1α and is induced under hypoxic conditions [13]. Secreted VEGF-A is involved in angiogenesis, tumor growth, and tumor metastasis [13,14]. VEGF-A is highly expressed in lung cancer tissues, and its overexpression is a poor prognostic factor [15]. Furthermore, VEGF-A increases the presence and function of myeloid-derived suppressor cells, regulatory T cells, and tumor-associated macrophages, which suppress anticancer immunity and inhibit cytotoxic T lymphocytes and dendritic cells [16]. Hence, VEGF-A promotes the development of an immunosuppressive tumor microenvironment, which may affect the therapeutic efficacy of anti-PD-1/PD-L1 antibodies.

The VEGF-A gene is located on chromosome 6p21.1 and consists of eight exons separated by seven introns [17]. The alternative splicing of VEGF-A mRNA from exons 5 to 8 produces different VEGF-A isoforms, such as VEGF_121_, VEGF_165_, VEGF_189_, and VEGF_206_ [18,19,20]. Of these, VEGF_121_ and VEGF_165_ are primarily secreted by tumor cells [21]. In a cancer mouse model with the overexpression of VEGF_121_ or VEGF_165_, VEGF_121_ promotes tumor growth, whereas VEGF_165_ suppresses it [22]. However, no studies have examined the association between the efficacy of anti-PD-1/PD-L1 antibody monotherapy and the levels of VEGF-A isoforms in the blood.

VEGF-A can be measured in both serum and plasma; however, because serum VEGF-A levels include VEGF-A pooled in the platelets, serum VEGF-A levels have been reported to be approximately 2–7 times higher than plasma levels [23,24]. Additionally, conflicting reports have demonstrated a relationship between the efficacy of anti-PD-1 antibody therapy in NSCLC and circulatory VEGF-A levels [25,26]. Shibaki et al. revealed that higher levels of VEGF-A in serum were associated with shorter survival [25], although Tiako et al. showed that there was no significant association between VEGF-A levels in plasma and efficacy in patients with NSCLC [26]. These data suggest that the usefulness of VEGF-A as a blood marker for predicting the efficacy of anti-PD-1/PD-L1 antibody therapy in patients with NSCLC depends on the sample type, such as serum or plasma.

Therefore, we investigated whether the association between the efficacy of anti-PD-1/PD-L1 antibody monotherapy and the circulatory levels of total VEGF-A (tVEGF-A) was dependent on sample types, such as serum and plasma, and compared the predictive value of tVEGF-A and its major isoforms, VEGF_121_ and VEGF_165_, for the efficacy of anti-PD-1/PD-L1 antibody monotherapy.

## 2. Materials and Methods

### 2.1. Study Population and Design

**Figure 1:**
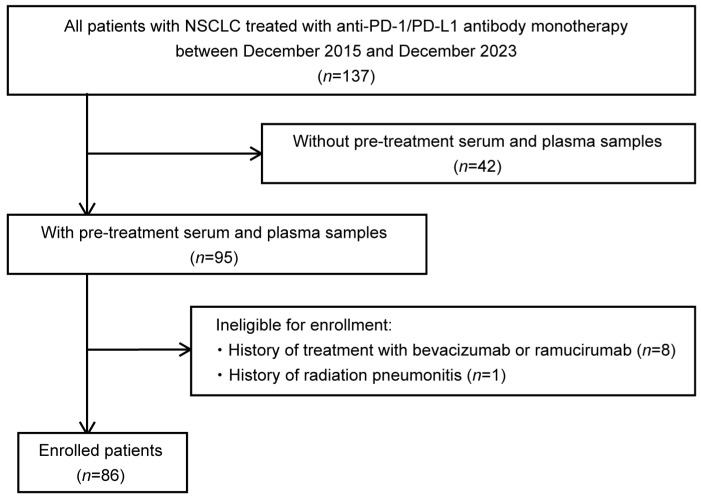
Flowchart of patient enrollment. This study included patients with non-small-cell lung cancer (NSCLC) treated with anti-programmed cell death 1(PD-1)/programmed cell death ligand 1(PD-L1) antibody monotherapy (nivolumab, pembrolizumab, or atezolizumab) at the Department of Respiratory Medicine, Hiroshima University Hospital, between December 2015 and December 2023, for whom serum and plasma samples were stored. After excluding eight patients who had a history of bevacizumab or ramucirumab prior to anti-PD-1/PD-L1 antibody administration and one patient who developed radiation pneumonitis just prior to the initiation of anti-PD-1/PD-L1 antibody monotherapy, 86 patients were finally included in the study. NSCLC, non-small-cell lung cancer; PD-1, programmed cell death 1; PD-L1, programmed cell death ligand 1.

This study screened 137 patients with NSCLC treated with anti-PD-1/PD-L1 antibody monotherapy (nivolumab, pembrolizumab, or atezolizumab) at the Department of Respiratory Medicine, Hiroshima University Hospital, between December 2015 and December 2023 (Figure 1). Forty-two patients without serum and plasma samples were excluded. Because the administration of bevacizumab and ramucirumab has been reported to cause fluctuations in circulatory VEGF-A [27,28,29], eight patients with a history of bevacizumab or ramucirumab before anti-PD-1/PD-L1 antibody administration were also excluded. Moreover, one patient who developed radiation pneumonitis immediately before the initiation of anti-PD-1/PD-L1 antibody monotherapy was excluded because VEGF-A levels may fluctuate owing to the development of pneumonitis [30]. Ultimately, 86 patients with serum and plasma samples were included in this study. This study was performed in accordance with the principles of the Declaration of Helsinki and approved by the Ethics Committee of Hiroshima University Hospital (E2004-0326-23, approved 7 August 2024). Written informed consent was obtained from all the participants.

### 2.2. Evaluations of the Objective Response Rate and Progression-Free Survival

Complete response (CR), partial response (PR), stable disease (SD), progressive disease (PD), and not evaluable (NE) were determined based on the Response Evaluation Criteria in Solid Tumors (RECIST) 1.1 [31]. The objective response rate (ORR) was defined as the proportion of patients who achieved CR or PR. Progression-free survival (PFS) was defined as the time from the start of each treatment until progression or death from any cause. Patients who failed to follow-up were censored on the date of their last known survival.

### 2.3. Measurement of tVEGF-A and Its Isoforms

Serum and plasma samples were collected prior to anti-PD-1/PD-L1 antibody administration and stored at −80 °C. Serum and plasma tVEGF-A levels were determined using an ELISA system developed by Shino-Test Corporation. Polystyrene microtiter plates were coated and incubated with 100 μL of anti-human VEGF-A polyclonal antibody (R&D Biosystems, Minneapolis, MN, USA) in PBS overnight at 4 °C. The plates were washed three times with PBS containing 0.05% Tween 20, and the remaining binding sites in the wells were blocked by incubating the plates for 2 h with 400 μL/well of PBS containing 0.5% casein. After the plates were washed, 100 μL of each dilution of the calibrator and samples (1:1 dilution in 0.2 mol/L Tris pH 8.5 and 0.15 mol/L sodium chloride containing 1% casein) was added to the wells. The plates were then incubated for 15 h at 25 °C. The plates were washed again and were incubated with 100 μL/well of peroxidase-conjugated anti-human VEGF-A monoclonal antibody (R&D Biosystems, Minneapolis, MN) for 2 h at 25 °C. After another washing step, chromogenic substrate 3,3′,5,5′-tetra-methylbenzidine (Dojindo Laboratories, Kumamoto, Japan) was added to each well. The reaction was terminated with sulfuric acid, and the absorbance at 450 nm was read using a microplate reader (Model 680, Bio-Rad, Irvine, CA, USA). VEGF_121_ and VEGF_165_ levels were measured using ELISA kits (Shino-Test, Kanagawa, Japan) [19].

### 2.4. Statistical Analysis

Values are expressed as a median (interquartile range [IQR]) unless stated otherwise. Differences among the groups were examined using the Fisher’s exact, Wilcoxon signed-rank, and Mann–Whitney U tests. Spearman’s rank correlation coefficient was calculated to evaluate the association between the levels of tVEGF-A and its isoforms. A receiver operating characteristic (ROC) curve analysis was performed to identify the optimal cut-off levels of tVEGF-A and its isoforms for predicting the objective response (CR or PR) to anti-PD-1/PD-L1 antibody monotherapy. The optimal cut-off level was determined by maximizing the sum of sensitivity plus specificity − 1. PFS was evaluated using a Kaplan–Meier analysis and the log-rank test. Median PFS intervals with a corresponding 95% confidence interval (CI) were calculated. Univariate and multivariate Cox proportional hazard models and logistic regression analyses were used to identify the independent predictors of PFS and the objective response for anti-PD-1/PD-L1 antibody monotherapy, respectively. Statistical significance was set at *p* < 0.05. All data analyses were performed using JMP statistical software version 17.0.0 (SAS Institute Inc., Cary, NC, USA).

## 3. Results

### 3.1. Patient Characteristics

The baseline characteristics of the patients are shown in Table 1. Of the 86 patients, the median age was 73 years (67–77), 60 (69.8%) were male, and 14 (16.3%) were NSCLC positive for driver oncogenes. PD-L1 TPS was ≥50% in 41 (47.7%), 1–49% in 18 (20.9%), <1% in 8 (9.3%), and unknown in 19 (22.1%). Anti-PD-1/PD-L1 antibody monotherapy was administered to 54 patients (62.8%) as a second- or later-line treatment.

The median observation period was 10.6 months (4.6–30.6). At the data cut-off in July 2024, progression or death from any cause was observed in 78 patients (90.7%). The treatment responses to anti-PD-1/PD-L1 antibody monotherapy in 86 patients were classified as CR in 4 (4.7%), PR in 16 (18.6%), SD in 17 (19.8%), PD in 36 (41.9%), and NE in 13 (15.1%). The ORR and median PFS of anti-PD-1/PD-L1 antibody monotherapy were 23.3% and 2.9 months (95% CI: 2.1–3.4), respectively.

### 3.2. Prediction of the Therapeutic Effect of Anti-PD-1/PD-L1 Antibody Monotherapy by Serum and Plasma tVEGF-A

**Figure 2:**
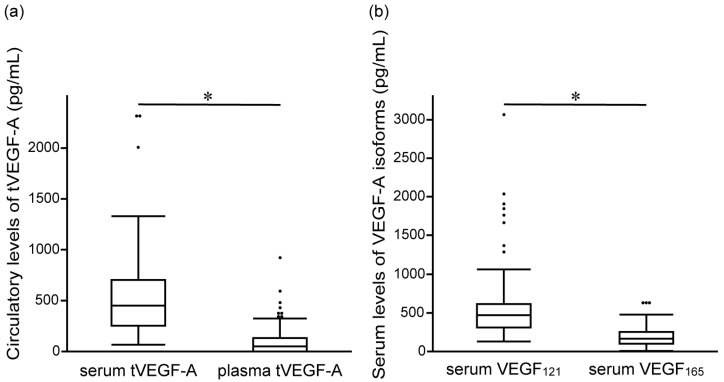
Comparison of baseline levels of (**a**) serum and plasma tVEGF-A, and (**b**) serum VEGF_121_ and VEGF_165_ before the initiation of anti-programmed cell death 1/programmed cell death ligand 1 antibody monotherapy. The serum levels of tVEGF-A are significantly higher than the plasma levels of tVEGF-A (452.9 pg/mL [interquartile range (IQR), 252.3–704.7] vs. 49.4 pg/mL [IQR, 0.0–131.6], *p* < 0.001) (**a**). The serum levels of VEGF_121_ are significantly higher than the serum levels of VEGF_165_ (466.4 pg/mL [IQR, 309.3–611.9] vs. 169.4 pg/mL [IQR, 98.8–251.8], *p* < 0.001) (**b**). The boxes represent the 25th to 75th percentiles; the solid lines within the boxes show the median values; the whiskers represent the 10th and 90th percentiles; the dots represent outliers. IQR, interquartile range; tVEGF, total vascular endothelial growth factor. * *p* < 0.001, using the Wilcoxon signed-rank test.

**Figure 3:**
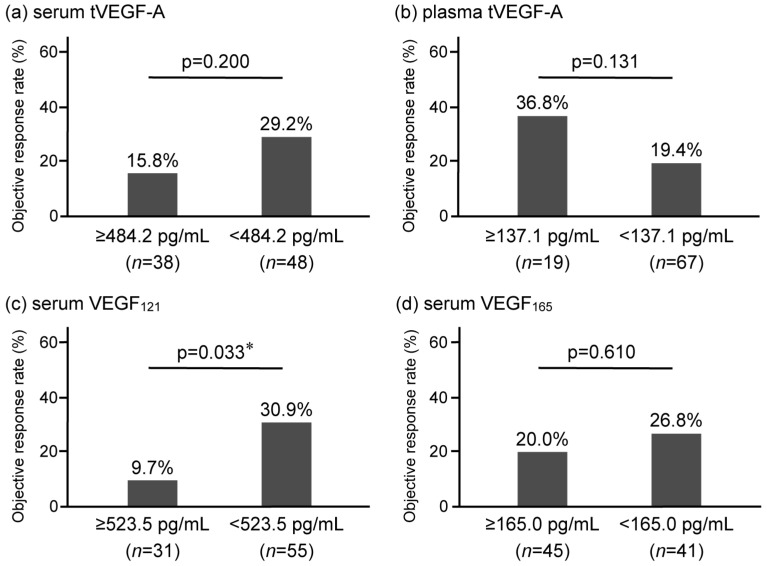
Comparison of the objective response rate (ORR) of anti-programmed cell death 1/programmed cell death ligand 1 antibody monotherapy in non-small-cell lung cancer stratified by baseline levels of (**a**) serum tVEGF-A, (**b**) plasma tVEGF-A, (**c**) serum VEGF_121_, and (**d**) serum VEGF_165_. The ORR is not significantly different for serum tVEGF-A (**a**) and plasma tVEGF-A (**b**). In contrast, the ORR is significantly lower in the high serum VEGF_121_ group (9.7% vs. 30.9, *p* = 0.033) (**c**) but not significantly different in serum VEGF_165_ (20.0% vs. 26.8%, *p* = 0.610) (**d**). ORR, objective response rate; tVEGF, total vascular endothelial growth factor. * *p* < 0.05, using the Fisher’s exact test.

**Figure 4:**
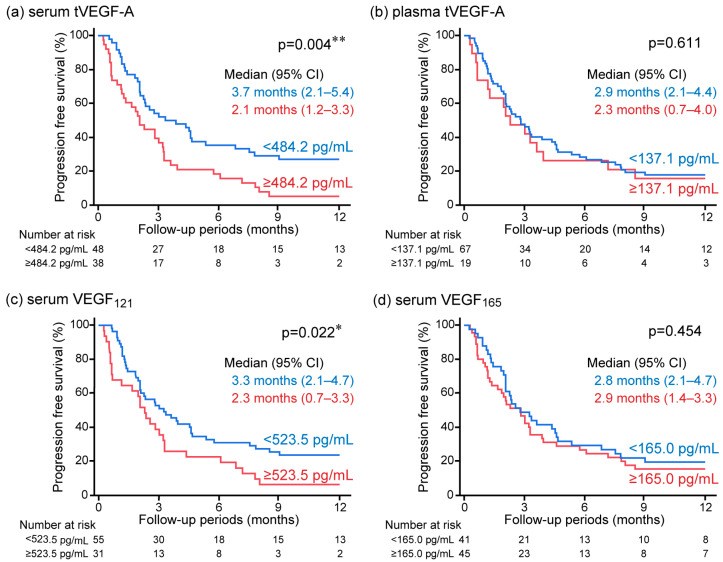
Kaplan–Meier analysis for progression-free survival (PFS) in anti-programmed cell death 1/programmed cell death ligand 1 antibody monotherapy in non-small-cell lung cancer stratified by baseline (**a**) serum tVEGF-A, (**b**) plasma tVEGF-A, (**c**) serum VEGF_121_, and (**d**) serum VEGF_165_. Patients with higher levels of serum tVEGF-A showed significantly shorter PFS than those with lower levels (**a**), but no significant difference in PFS was observed when stratified by plasma tVEGF-A (**b**). Also, patients with higher levels of serum VEGF_121_ showed significantly shorter PFS than those with lower levels (**c**), but there was no significant difference in PFS when evaluated by serum VEGF_165_ (**d**). CI, confidence interval; PFS, progression-free survival; tVEGF, total vascular endothelial growth factor. * *p* < 0.05 and ** *p* < 0.01 using the log-rank test.

The serum and plasma levels of tVEGF-A were measured. Serum tVEGF-A levels were significantly higher than plasma levels (452.9 pg/mL [252.3–704.7] vs. 49.4 pg/mL [0.0–131.6], *p* < 0.001) (Figure 2a, Supplementary Table S1). Serum and plasma tVEGF-A levels were positively correlated (ρ = 0.502, *p* < 0.001) (Supplementary Figure S1a). The ROC curve analysis revealed that the optimal cut-off levels for predicting the objective response to anti-PD-1/PD-L1 antibody monotherapy were 484.2 pg/mL for serum tVEGF-A (area under the curve [AUC] = 0.54 [95% CI: 0.40–0.68], specificity = 48.5%, sensitivity = 70.0%) and 137.1 pg/mL for plasma tVEGF-A (AUC = 0.54 [95% CI: 0.39–0.68], specificity = 81.8%, sensitivity = 35.0%) (Supplementary Figure S2a,b). There was no significant difference in the ORR between the groups stratified by serum and plasma tVEGF-A cut-off levels (Figure 3a,b). Conversely, the Kaplan–Meier analysis showed that PFS was significantly shorter in patients with higher levels of serum tVEGF-A than in those with lower levels (median PFS 2.1 months [95% CI: 1.2–3.3] vs. 3.7 months [95% CI: 2.1–5.4], *p* = 0.004), but there was no significant difference in PFS between patients stratified by the cut-off levels of plasma tVEGF-A (median PFS 2.3 months [95% CI: 0.7–4.0] vs. 2.9 months [95% CI: 2.1–4.4], *p* = 0.611) (Figure 4a,b). The univariate Cox proportional hazards model revealed that serum tVEGF-A levels, a history of chronic obstructive pulmonary disease (COPD), an immune checkpoint inhibitor (ICI) treatment line, and the ICI agent were significant predictors of PFS (Table 2). Furthermore, the multivariate Cox proportional hazards model (model 1) revealed that serum tVEGF-A levels (≥484.2 pg/mL) were independent predictors of shorter PFS when adjusted for a history of COPD, ICI treatment line, and ICI agent (Table 2).

### 3.3. Prediction of the Therapeutic Effect of Anti-PD-1/PD-L1 Antibody Monotherapy by Serum VEGF-A Isoforms

This study additionally measured VEGF_121_ and VEGF_165_ levels using serum samples, as only the serum levels of tVEGF-A, not the plasma levels, were used to stratify PFS. The serum levels of VEGF_121_ were significantly higher than the serum levels of VEGF_165_ (466.4 pg/mL [309.3–611.9] vs. 169.4 pg/mL [98.8–251.8], *p* < 0.001) (Figure 2b, Supplementary Table S1). The serum levels of VEGF_121_ and VEGF_165_ were positively correlated with the serum levels of tVEGF-A (ρ = 0.607, *p* < 0.001 and ρ = 0.865, *p* < 0.001, respectively) (Supplementary Figure S1b,c). The ROC curve analysis revealed that the optimal cut-off levels for predicting the objective response to anti-PD-1/PD-L1 antibody monotherapy were 523.5 pg/mL for serum VEGF_121_ (AUC = 0.61 [95% CI: 0.47–0.73], specificity = 42.4%, sensitivity = 85.0%) and 165.0 pg/mL for serum VEGF_165_ (AUC = 0.50 [95% CI: 0.36–0.65], specificity = 54.6%, sensitivity = 55.0%) (Supplementary Figure S2c,d).

The ORR was significantly lower in patients with higher levels of serum VEGF_121_ (≥523.5 pg/mL) than in those without (9.7% vs. 30.9%, *p* = 0.033), although there was no significant difference in the ORR between patients with and without VEGF_165_ serum levels higher than 165.0 pg/mL (20.0% vs. 26.8%, *p* = 0.610) (Figure 3c,d). Univariate and multivariate logistic regression analyses revealed that, among circulatory tVEGF-A and its isoforms, only higher levels of serum VEGF_121_ were independently and significantly associated with a failure to achieve the objective response (Table 3).

The Kaplan–Meier analysis also showed a significantly shorter PFS in the group with higher levels of serum VEGF_121_ than in the group without (median PFS 2.3 months [95% CI: 0.7–3.3] vs. 3.3 months [95% CI: 2.1–4.7] months, *p* = 0.022), but not in the group with and without higher levels of serum VEGF_165_ (median PFS 2.9 months [95% CI: 1.4–3.3] vs. 2.8 months [95% CI: 2.1–4.7] months, *p* = 0.454) (Figure 4c,d). The univariate Cox proportional hazards model revealed that serum VEGF_121_ levels were significant predictors of PFS (Table 2). A positive correlation was observed between the serum levels of tVEGF-A and VEGF_121_; therefore, the association between PFS and serum VEGF_121_ was analyzed in a multivariate Cox proportional hazards model not including serum tVEGF-A. The multivariate Cox proportional hazards model (model 2) revealed that higher levels of serum VEGF_121_ were an independent predictor of shorter PFS when adjusted for a history of COPD, ICI treatment line, and ICI agent (Table 2).

## 4. Discussion

In this study, serum and plasma tVEGF-A levels were examined to predict the efficacy of anti-PD-1/PD-L1 antibody monotherapy in patients with NSCLC. Higher levels of tVEGF-A in serum, but not in plasma, were significantly associated with a shorter PFS. Furthermore, among the serum levels of tVEGF-A and its isoforms, higher levels of serum VEGF_121_ were useful for predicting a lower ORR and shorter PFS in patients treated with anti-PD-1/PD-L1 antibody monotherapy.

This study demonstrated that serum samples were suitable for measuring tVEGF-A levels to stratify the PFS of patients receiving anti-PD-1/PD-L1 antibody monotherapy. Consistent with our results, high serum tVEGF-A levels have been reported to be associated with shorter PFS in patients with NSCLC who are elderly or have poor PS [25]; however, these associations have not been shown in other studies using plasma samples [26]. This discrepancy in the association between the efficacy of anti-PD-1/PD-L1 antibodies and serum or plasma tVEGF-A levels is potentially caused by platelet-derived VEGF-A in the serum. First, in circulation, most VEGF-A is pooled in the alpha granules of platelets, and VEGF-A is released from platelets, particularly when platelets are activated by several factors, including blood coagulation [32,33]. When serum samples are obtained, VEGF-A is released from platelets owing to blood coagulation in the serum collection tubes [33]. Accordingly, serum VEGF-A levels have been reported to be approximately two to seven times higher than plasma VEGF-A levels [23,24]. It has also been shown that the VEGF-A content of platelets increases with tumor progression, and much of the serum VEGF-A in patients with cancer is thought to be derived from VEGF-A pooled in platelets [10,11]. Secondly, VEGF-A released from activated platelets by tumor cells plays a role in promoting tumor progression and metastasis [34,35]. Additionally, VEGF-A promotes the development of an immunosuppressive tumor microenvironment associated with resistance to anti-PD-1/PD-L1 antibody therapy [16]. These data suggest that the measurement of VEGF-A levels pooled in platelets is needed to predict the efficacy of anti-PD-1/PD-L1 antibody therapy, and therefore, serum tVEGF-A could be a predictive biomarker for efficacy by reflecting the amount of VEGF-A in platelets in this study.

This study also showed that higher levels of serum VEGF_121_ were significantly and independently associated with a lower ORR and shorter PFS in patients treated with anti-PD-1/PD-L1 antibody monotherapy, although high levels of serum tVEGF-A were associated with shorter PFS but not the ORR. Additionally, VEGF_165_ serum levels could not be used to stratify PFS or the ORR. VEGF_121_ promotes tumor angiogenesis and increases vascular permeability around the tumor [22,36,37,38]. Moreover, VEGF_121_ promotes lymphangiogenesis in the sentinel lymph nodes of NSCLC cells [39]. Furthermore, in various types of cancer, including lung cancer, high expression of VEGF_121_ evaluated using tumor samples has been shown to be associated with poor prognosis [40,41,42]. In contrast, VEGF_165_ inhibits tumor growth by normalizing tumor blood vessels and reducing the hypoxic state of the tumors. VEGF_165_ has an NRP-binding domain and can bind to NRP1, although VEGF_121_ cannot accelerate NRP1 signaling [43]. The interaction between VEGF_165_ and NRP1 recruits NRP1-expressing monocytes (NEMs) to newly formed blood vessels [43]. NEMs produce molecules that contribute to the stabilization of tumor blood vessels (such as transforming growth factor-β, platelet-derived growth factor-B, and stromal cell-derived factor-1) and chemokines with anti-tumor activity (C-C motif chemokine ligand [CCL]2, CCL4, CCL5, C-X-C motif chemokine ligand [CXCL]9, CXCL10, etc.), thereby inhibiting tumor growth [44]. Therefore, the use of VEGF_121_, which is involved in tumor growth more specifically than tVEGF-A, including VEGF_165_, could stratify both the PFS and ORR of anti-PD-1/PD-L1 antibody treatment.

This study has several limitations. First, this was a retrospective study with a limited sample size. Second, the study included patients who received anti-PD-1/PD-L1 antibody monotherapy from the second-line treatment onwards. Currently, ICI alone or in combination with chemotherapy is administered as the first-line therapy for the treatment of advanced-stage NSCLC in most cases. To further investigate the usefulness of VEGF_121_, it is necessary to confirm the results of this study in a prospective cohort of patients with NSCLC who received anti-PD-1/PD-L1 antibody therapy as the first-line treatment. Additionally, to overcome ICI resistance in patients with higher levels of serum VEGF_121_, our future perspective is that the efficacy of the combination therapy with molecular targeted therapies for VEGF-A or VEGFR needs to be evaluated. Third, the AUC value of serum VEGF_121_ was 0.61, which is not sufficiently high. To enhance its predictive accuracy, a combination with other predictive markers may be necessary. Fourth, VEGF-A levels may have fluctuated due to the influence of the fasting state and time of day [45,46], as the conditions for obtaining blood samples were not unified with considering these factors in this study.

## 5. Conclusions

In patients with NSCLC who received anti-PD-1/PD-L1 antibody monotherapy, high serum tVEGF-A levels, but not plasma levels, were significantly associated with a shorter PFS. Furthermore, as the focus was on the VEGF-A isoforms VEGF_121_ and VEGF_165_, only VEGF_121_ could be used as a predictive marker for the efficacy of anti-PD-1/PD-L1 antibody monotherapy, and high serum VEGF_121_ levels were associated with a low ORR and shorter PFS. Therefore, VEGF-A levels, particularly VEGF_121_, measured by serum levels, could be a predictive biomarker for the efficacy of anti-PD-1/PD-L1 antibody monotherapy.

## Figures and Tables

**Table 1:** Baseline characteristics.

	All Patients (*n* = 86)	CR/PR (*n* = 20)	SD/PD/NE (*n* = 66)	*p*-Value
Age, years	73 (67–77)	70 (68–79)	73 (66–77)	0.705
Sex				0.163
Male, *n* (%)	60 (69.8)	11 (55.0)	49 (74.2)	
Female, *n* (%)	26 (30.2)	9 (45.0)	17 (25.8)	
Smoking history, pack-years ^†^	50.0 (22.5–60.0)	56.3 (30.0–79.5)	49.8 (16.0–60.0)	0.242
BMI	21.3 (19.3–23.2)	21.2 (19.7–23.1)	21.3 (19.0–23.2)	0.759
PS				0.221
0–1, *n* (%)	68 (79.1)	18 (90.0)	50 (75.8)	
≥2, *n* (%)	18 (20.9)	2 (10.0)	16 (24.2)	
History of COPD				0.427
+, *n* (%)	31 (36.0)	9 (45.0)	22 (33.3)	
–, *n* (%)	55 (64.0)	11 (55.0)	44 (66.7)	
Previous thoracic RT				0.405
+, *n* (%)	26 (30.2)	4 (20.0)	22 (33.3)	
–, *n* (%)	60 (69.8)	16 (80.0)	44 (66.7)	
Stage				0.022 *
III, *n* (%)	8 (9.3)	5 (25.0)	3 (4.5)	
IV, *n* (%)	50 (58.1)	11 (55.0)	39 (59.1)	
Recurrence, *n* (%)	28 (32.6)	4 (20.0)	24 (36.4)	
Histological type				1.000
Squamous, *n* (%)	13 (15.1)	3 (15.0)	10 (15.2)	
Non-Squamous, *n* (%)	73 (84.9)	17 (85.0)	56 (84.8)	
Driver oncogene ^‡^				0.505
positive, *n* (%)	14 (16.3)	2 (10.0)	12 (18.2)	
negative, *n* (%)	72 (83.7)	18 (90.0)	54 (81.8)	
PD-L1 TPS				0.122
≥50%, *n* (%)	41 (47.7)	14 (70.0)	27 (40.9)	
1–49%, *n* (%)	18 (20.9)	3 (15.0)	15 (22.7)	
<1%, *n* (%)	8 (9.3)	0 (0.0)	8 (12.1)	
unknown, *n* (%)	19 (22.1)	3 (15.0)	16 (24.2)	
ICI treatment line				0.070
1st, *n* (%)	32 (37.2)	11 (55.0)	21 (31.8)	
2nd or later, *n* (%)	54 (62.8)	9 (45.0)	45 (68.2)	
ICI agent				0.009 **
Anti-PD-1 antibody, *n* (%)	69 (80.2)	20 (100.0)	49 (74.2)	
Anti-PD-L1 antibody, *n* (%)	17 (19.8)	0 (0.0)	17 (25.8)	

Data are presented as a median (interquartile range) unless stated otherwise. BMI, body mass index; COPD, chronic obstructive pulmonary disease; CR, complete response; ICI, immune checkpoint inhibitor; NE, not evaluable; PD, progressive disease; PD-1, programmed cell death 1; PD-L1, programmed cell death ligand 1; PR, partial response; PS, performance status; RT, radiotherapy; SD, stable disease; TPS, tumor proportion score. ^†^ There are missing data for one patient. ^‡^ Driver oncogenes included 12 patients with epidermal growth factor receptor gene mutations and 2 patients with mesenchymal–epithelial transition exon 14 skipping. * *p* < 0.05 and ** *p* < 0.01, comparison between CR/PR and SD/PD/NE using the Mann–Whitney U test or Fisher’s exact test.

**Table 2:** Univariate and multivariate Cox proportional hazards model for predicting progression-free survival in patients with non-small-cell lung cancer treated with anti-programmed cell death 1(PD-1)/programmed cell death ligand 1(PD-L1) antibody monotherapy.

Variables	Univariate Analysis			Multivariate Analysis (Model 1)			Multivariate Analysis (Model 2)		
	HR	95% CI	*p*-Value	HR	95% CI	*p*-Value	HR	95% CI	*p*-Value
Age, ≥75	0.788	0.488–1.273	0.330						
Sex, male	1.587	0.927–2.717	0.092						
Smoking history, pack-years	0.996	0.989–1.003	0.236						
BMI	0.993	0.930–1.058	0.829						
PS, ≥2	1.231	0.705–2.149	0.466						
History of COPD	0.555	0.334–0.922	0.023 *	0.821	0.478–1.410	0.475	0.800	0.462–1.385	0.425
Previous thoracic RT	0.987	0.596–1.632	0.958						
Histological type, squamous	0.805	0.423–1.534	0.510						
Driver oncogene, positive	1.521	0.831–2.784	0.174						
PD-L1 TPS, ≥50%	0.722	0.450–1.159	0.178						
ICI treatment line, 1st	0.577	0.351–0.948	0.030 *	0.580	0.330–1.017	0.057	0.696	0.404–1.198	0.191
ICI agent, anti-PD-1 antibody	0.303	0.169–0.545	<0.001 ***	0.317	0.166–0.606	<0.001 ***	0.317	0.164–0.613	<0.001 ***
Serum tVEGF-A, ≥484.2 pg/mL	1.952	1.220–3.124	0.005 **	2.511	1.496–4.212	<0.001 ***			
Plasma tVEGF-A, ≥137.1 pg/mL	1.155	0.661–2.015	0.613						
Serum VEGF_121_, ≥523.5 pg/mL	1.731	1.074–2.790	0.024 *				1.967	1.167–3.314	0.011 *
Serum VEGF_165_, ≥165.0 pg/mL	1.194	0.749–1.904	0.457						

Two multivariate Cox proportional hazards models were analyzed because a positive correlation was observed between the serum levels of tVEGF-A and VEGF_121_. BMI, body mass index; CI, confidence interval; COPD, chronic obstructive pulmonary disease; HR, hazard ratio; ICI, immune checkpoint inhibitor; PD-1, programmed cell death 1; PD-L1, programmed cell death ligand 1; PS, performance status; RT, radiotherapy; TPS, tumor proportion score; tVEGF, total vascular endothelial growth factor. * *p* < 0.05, ** *p* < 0.01 and *** *p* < 0.001, Cox proportional hazards models.

**Table 3:** Univariate and multivariate logistic regression analyses for predicting the objective response in patients with non-small-cell lung cancer treated with anti-programmed cell death 1(PD-1)/programmed cell death ligand 1(PD-L1) antibody monotherapy.

Variables	Univariate Analysis			Multivariate Analysis		
	OR	95% CI	*p*-Value	OR	95% CI	*p*-Value
Age, ≥75	1.342	0.479–3.698	0.570			
Sex, male	0.424	0.149–1.215	0.109			
Smoking history, pack-years	1.005	0.992–1.019	0.432			
BMI	1.006	0.867–1.165	0.932			
PS, ≥2	0.347	0.052–1.387	0.145			
History of COPD	1.636	0.581–4.549	0.346			
Previous thoracic RT	0.500	0.131–1.559	0.242			
Histological type, squamous	0.988	0.205–3.679	0.987			
Driver oncogene, positive	0.500	0.073–2.068	0.364			
PD-L1 TPS, ≥50%	3.370	1.193–10.551	0.021 *	2.645	0.864–8.845	0.089
ICI treatment line, 1st	2.619	0.947–7.451	0.064			
ICI agent, anti-PD-1 antibody	Not available ^†^	Not available ^†^	0.001 **	Not available ^†^	Not available ^†^	0.005 **
Serum tVEGF-A, ≥484.2 pg/mL	0.455	0.146–1.284	0.139			
Plasma tVEGF-A, ≥137.1 pg/mL	2.423	0.774–7.337	0.126			
Serum VEGF_121_, ≥523.5 pg/mL	0.239	0.052–0.799	0.019 *	0.231	0.049–0.819	0.022 *
Serum VEGF_165_, ≥165.0 pg/mL	0.682	0.244–1.862	0.454			

BMI, body mass index; CI, confidence interval; COPD, chronic obstructive pulmonary disease; ICI, immune checkpoint inhibitor; OR, odds ratio; PD-1, programmed cell death 1; PD-L1, programmed cell death ligand 1; PS, performance status; RT, radiotherapy; TPS, tumor proportion score; tVEGF, total vascular endothelial growth factor. ^†^ OR and 95% CI could not be calculated because only one group responded to anti-PD-1/PD-L1 antibody monotherapy. * *p* < 0.05 and ** *p* < 0.01, logistic regression analysis.

## Data Availability

The raw data supporting the conclusions of this article will be made available by the authors on request.

## Supplementary Materials

The following supporting information can be downloaded at: https://www.mdpi.com/article/10.3390/cancers17040572/s1. Figure S1: Association between circulatory levels of tVEGF-A and VEGF-A isoforms; Figure S2: Receiver operating characteristic (ROC) curve analysis for predicting the complete response or partial response to anti-programmed cell death 1/programmed cell death ligand 1 antibody monotherapy; Table S1: Baseline levels of circulatory tVEGF-A and VEGF-A isoforms before the initiation of anti-programmed cell death 1/programmed cell death ligand 1 antibody monotherapy.

## Abbreviations

The following abbreviations are used in this manuscript:

AUC	area under the curve
CCL	C-C motif chemokine ligand
CI	confidence interval
COPD	chronic obstructive pulmonary disease
CR	complete response
CXCL	C-X-C motif chemokine ligand
ELISA	enzyme-linked immunosorbent assay
ICI	immune checkpoint inhibitor
IQR	interquartile range
NSCLC	non-small-cell lung cancer
NE	not evaluable
NRP	neuropilin
ORR	objective response rate
PD-1	programmed cell death 1
PD-L1	programmed cell death ligand 1
PFS	progression-free survival
PR	partial response
PD	progressive disease
RECIST	Response Evaluation Criteria in Solid Tumors
ROC	receiver operating characteristic
SD	stable disease
tVEGF	total vascular endothelial growth factor
TPS	tumor proportion score
VEGF	vascular endothelial growth factor
VEGFR	vascular endothelial growth factor receptor
