# Supplementary material for: Predictive Value of Circulatory Total VEGF-A and VEGF-A Isoforms for the Efficacy of Anti-PD-1/PD-L1 Antibodies in Patients with Non-Small-Cell Lung Cancer
Source: Cancers (Basel). 2025 Feb 7;17(4):572. doi: 10.3390/cancers17040572 (PMC11853576; doi:10.3390/cancers17040572)
Supplement: Supplementary file 1 [file cancers-17-00572-s001.zip › cancers-3443992-supplementary.pdf]

Supplementary Figure S1. Association between circulatory levels of tVEGF-A and VEGF-A isoforms.

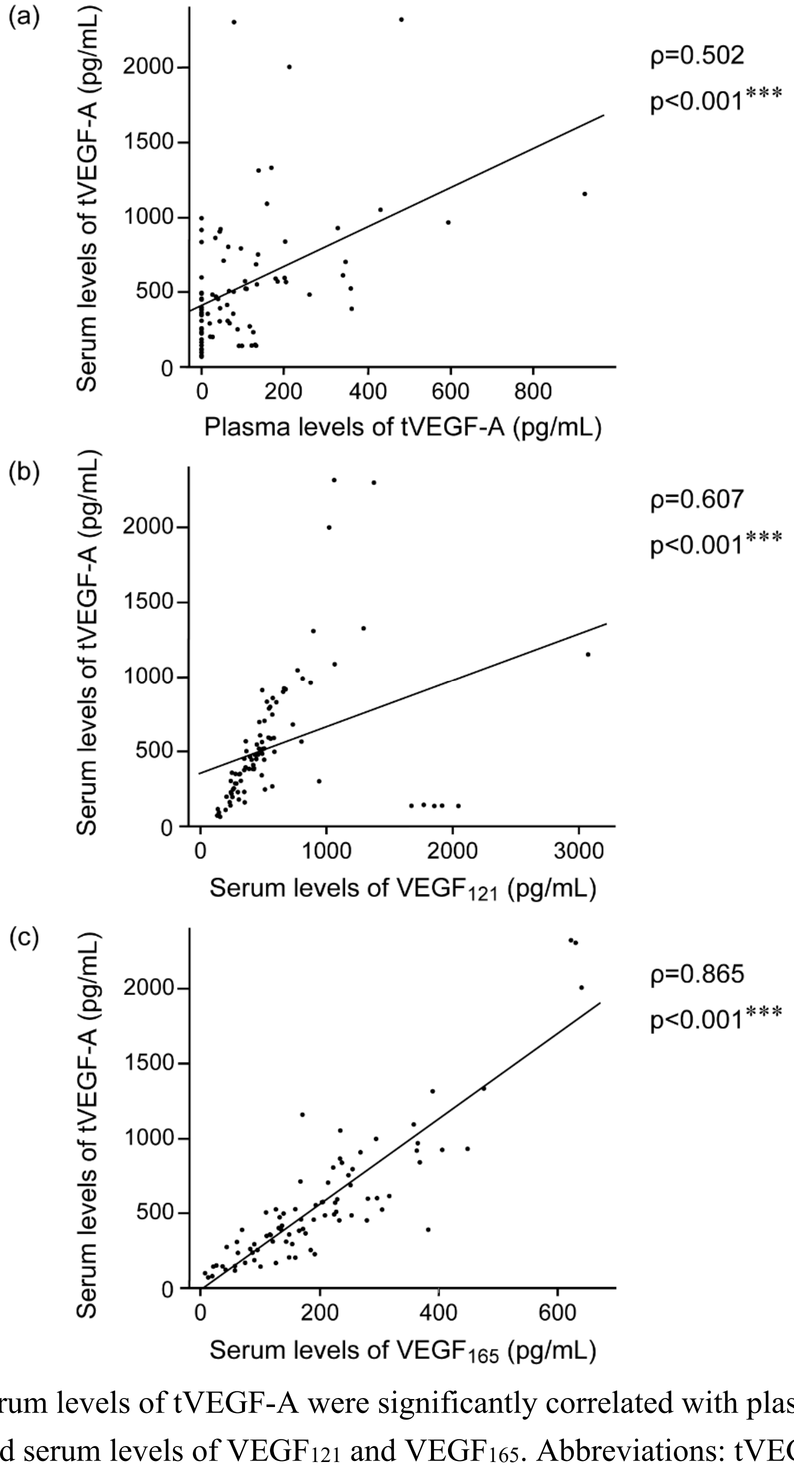

The serum levels of tVEGF-A were significantly correlated with plasma levels of tVEGF-A and serum levels of $VEGF_{121}$ and $VEGF_{165}$. Abbreviations: tVEGF, total vascular endothelial growth factor. $^{***}p < 0.001$ using the Spearman's rank correlation coefficient.

Supplementary Figure S2. Receiver operating characteristic (ROC) curve analysis for predicting the complete response or partial response to anti-programmed cell death 1/programmed cell death ligand 1 antibody monotherapy.

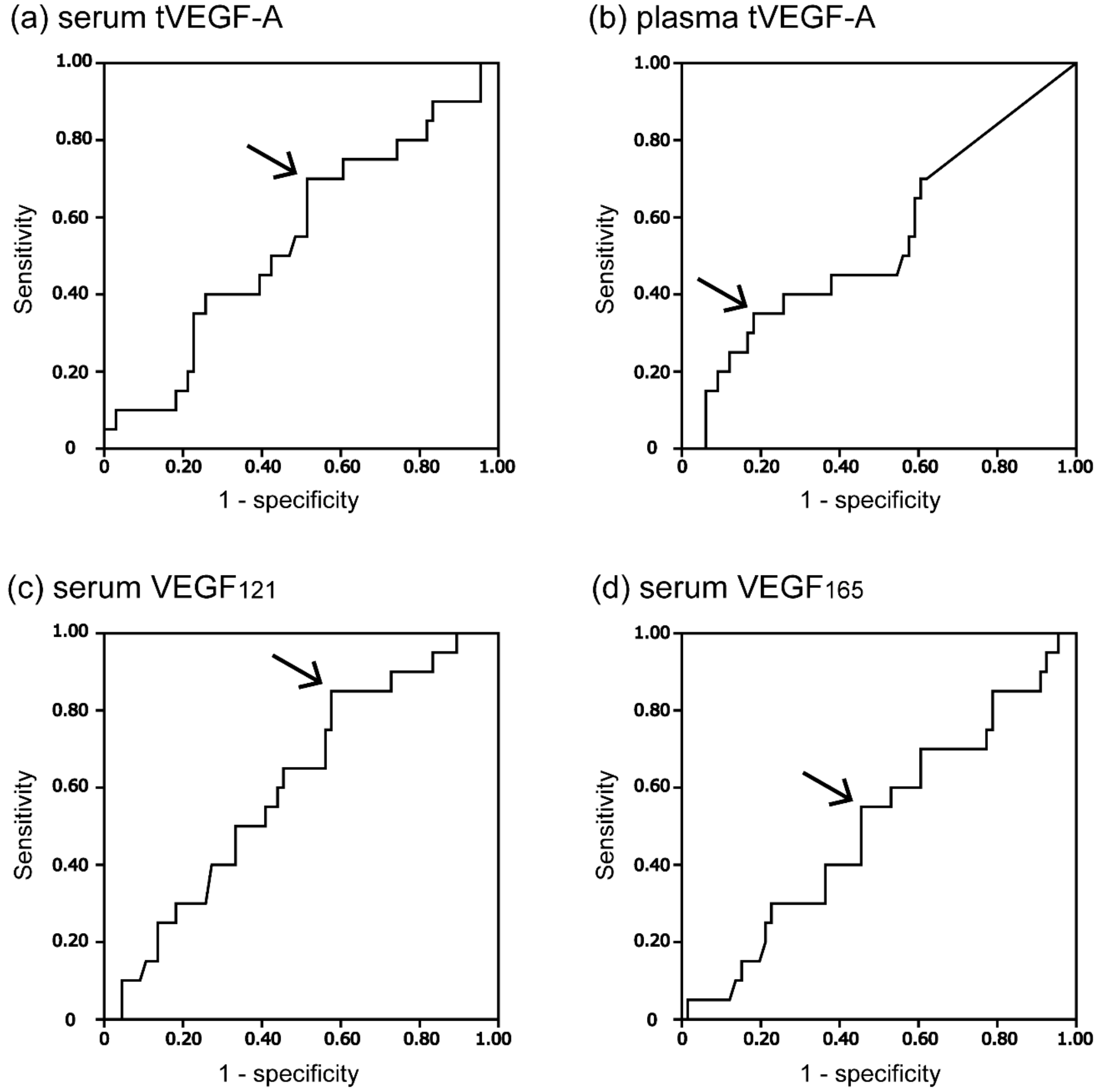

Values in parenthesis indicate 95% confidence interval (CI) for the area under the curve (AUC). ROC curve analysis revealed that the optimal cut-off levels were 484.2 pg/mL for serum total vascular endothelial growth factor (tVEGF)-A (AUC = 0.54 [0.40–0.68], specificity = 48.5%, sensitivity = 70.0%), 137.1 pg/mL for plasma tVEGF-A (AUC = 0.54 [0.39–0.68], specificity = 81.8%, sensitivity = 35.0%), 523.5 pg/mL for serum $VEGF_{121}$ (AUC = 0.61 [0.47–0.73], specificity = 42.4%, sensitivity = 85.0%), and 165.0 pg/mL for serum $VEGF_{165}$ (AUC = 0.50 [0.36–0.65], specificity = 54.6%, sensitivity = 55.0%).

Supplementary Table S1. Baseline levels of circulatory tVEGF-A and VEGF-A isoforms before the initiation of anti-programmed cell death 1/programmed cell death ligand 1 antibody monotherapy.

	**All patients (*n* = 86)**	**CR/PR (*n* = 20)**	**SD/PD/NE (*n* = 66)**	**p-value***
Serum tVEGF-A	452.9 (252.3–704.7)	420.2 (239.8–658.3)	456.2 (269.3–720.9)	0.550
Plasma tVEGF-A	49.4 (0.0–131.6)	39.2 (0.0–192.8)	57.2 (0.0–129.6)	0.561
Serum $VEGF_{121}$	466.4 (309.3–611.9)	409.2 (262.3–506.7)	483.6 (315.7–686.1)	0.151
Serum $VEGF_{165}$	169.4 (98.8–251.8)	161.8 (91.0–264.3)	171.1 (107.0–248.8)	0.951

Data are presented as median (interquartile range). CR, complete response; NE, not evaluable; PD, progressive disease; PR, partial response; SD, stable disease; tVEGF, total vascular endothelial growth factor.

* Comparison between CR/PR and SD/PD/NE using the Mann–Whitney *U* test.
